# The Oropharyngeal Morphology in the Semiaquatic Giant Asian Pond Turtle, *Heosemys grandis*, and Its Evolutionary Implications

**DOI:** 10.1371/journal.pone.0046344

**Published:** 2012-09-28

**Authors:** Monika Lintner, Anton Weissenbacher, Egon Heiss

**Affiliations:** 1 Department of Integrative Zoology, University of Vienna, Vienna, Austria; 2 Zoo Vienna, Vienna, Austria; 3 Department of Biology, University of Antwerp, Antwerp, Belgium; Monash University, Australia

## Abstract

The oropharynx as a functional entity plays a fundamental role in feeding. Transitions from aquatic to terrestrial lifestyles in vertebrates demanded major changes of the oropharynx for the required adaptations to a different feeding environment. Extant turtles evolved terrestrial feeding modes in three families (testudinids, emydids, geoemydids)–independently from other amniotes–and are therefore important model organisms to reconstruct morpho-functional changes behind aquatic-terrestrial transitions. In this study we hypothesized that the oropharyngeal morphology in semiaquatic turtles of the geoemydid family shows parallels to testudinids, the only purely terrestrial extant lineage. We provide an in-depth description of the oropharynx in the semiaquatic geoemydid *Heosemys grandis* by using a combination of micro computed tomography (micro-CT) and subsequent digital *in situ* 3-D reconstruction, scanning electron microscopy (SEM), and histology. We show that *H. grandis* has a large tongue with rough papillose surface and well-developed lingual muscles. The attachment sites of the lingual muscles on the hyolingual skeleton and their courses within the tongue are nearly identical with testudinids. The hyolingual skeleton itself is mainly cartilaginous and shows distinct–but compared to testudinids rather small–anterior extensions of the hyoid body and hypoglossum. Oral glands are well developed in *H. grandis* but are smaller and simpler than in testudinids. Similarly, oropharyngeal keratinization was minimal and found only in the anterior palate, regions close to the beak, and tongue tip. We conclude that *H. grandis* shows distinct oropharyngeal morpho-functional adaptations for a terrestrial lifestyle but still retains characters typical for aquatic forms. This makes this species an important example showing the oropharyngeal adaptations behind aquatic-terrestrial transitions in turtles.

## Introduction

The uptake of food is crucial for all animal life, and adaptations of the feeding biology to specific environments were essential for vertebrate evolution [1]. One of the most important steps in vertebrate evolution was the transition from aquatic to terrestrial forms. This transition required major changes in almost every organ system, including the morphologically and functionally highly integrated feeding mechanism [2]. Modern turtles (crown group, see [3]) have evolved a terrestrial lifestyle including terrestrial feeding, independently to other amniotes and still show the whole range from fully aquatic to fully terrestrial forms. This makes them important model organisms to reconstruct the morpho-functional mechanisms behind aquatic-terrestrial transitions in turtles and vertebrates in general.

In vertebrates, the oropharynx plays a major role in a variety of functions, including feeding [4]. While most aquatic turtles (and vertebrates in general) use an ingestion mode based on rapid oropharyngeal volume expansion to create inertial or compensatory suction [5], this strategy does not work on land due to the low viscosity and density of air compared to water [6]. On land, turtles ingest food by using their jaws (“jaw prehension”) or their tongue (“tongue prehension”) and use cyclical lingual movements for further intraoral transport [7]–[13]. The evolution of a movable tongue as part of the feeding system was therefore one of the key innovations behind aquatic-terrestrial transitions, providing a useful tool to exploit food resources in the new environment. Together with the development of a movable tongue, the increase in oropharyngeal glandular tissue is important for terrestrial feeding [14]–[18]. As a lubricating medium mucus plays an important role in many functions associated with terrestrial feeding [6]. Accordingly, aquatic turtles show only simple oropharyngeal glands, namely goblet cells [16], [19]–[25], while terrestrial turtles have large, complexly arranged glandular entities that are tubular, branched or alveolar [10], [14]–[16], [18], [20]. Similarly, keratinization of the oral mucosa provides protection from abrasion or dehydration in arid environments and is generally assumed to be better developed in terrestrial turtles [26]. Terrestrial lifestyles and food uptake independent from water evolved in only three (out of 14) families of extant turtles: in emydids (New World pond turtles), geoemydids (Old World pond turtles) and testudinids (tortoises) [3], [27]. While all testudinids are exclusively terrestrial, emydids and geoemydids contain a range from fully aquatic to mainly terrestrial along with many intermediate forms. Phylogenetic studies suggest a common ancestor of these three families and pool them together to the monophyletic superfamily Testudinoidea [3]. The common ancestor of Testudinoidea again was aquatic and consequently, terrestrial evolution probably happened independently in these three lineages. Unfortunately, only limited knowledge is available on the oropharyngeal morphology in members of these three families, but would be important to deduce morpho-functional changes in the oropharyngeal system behind aquatic terrestrial transitions in turtles. The present study was designed to provide a detailed description of the oropharynx in the geoemydid Giant Asian pond turtle *Heosemys grandis* which displays a semiaquatic lifestyle with tendency towards terrestrial environments [27]. We therefore hypothesize that it shows parallels to testudinid turtles in its oropharyngeal morphology. Specifically, we expect a well-developed tongue with large papillae on its dorsal lingual surface and a lingual myo-skeletal system comparable with testudinids. Similarly, oropharyngeal glands and keratinization in the oral mucosa are both hypothesized to be well developed and to reach the same amount as in testudinids. Alternatively, *H. grandis* could lack the above-described features by having developed other morpho-functional strategies for terrestrial feeding.

This study will provide important information on the oropharyngeal system in turtles in general and on the oropharyngeal changes behind terrestrial evolution in geoemydid and testudinid turtles in particular. A better understanding of the evolutionary mechanisms and patterns behind aquatic-terrestrial transitions can not only help reconstruct the history of tetrapods, but also improve our knowledge on the mechanisms by which major evolutionary transitions are accomplished [13].

## Materials and Methods

The Giant Asian Pond Turtle, *Heosemys grandis*, [28] lives in or close to water, including rivers, swamps, lakes, creeks, and ponds, from sea level up into the mountains. The home range of *H. grandis* includes southern Myanmar, Thailand, southern Cambodia, southern Vietnam, and peninsular Malaysia [27], [29]. In the literature this turtle is a true omnivore, eating aquatic and terrestrial plants, dead animals, insects, amphibian larvae, earthworms, and snails [27].

For the present study we used six subadult (two years old) *H. grandis* with a carapace length ranging from 140 to 152 mm and a weight from 413 to 430 g. The animals were all captive bred and obtained from a local breeder. The turtles were kept in an aquaterrarium with 150×100 cm ground area at a 12 h dark, 12 h light cycle. The tank contained bark mulch (5 cm high) as substrate, cork bark pieces as hiding places, and a low basin (40×100×7 cm) filled with water which was permanently filtered by an external aquarium filter. The animals were fed with a variety of vegetables, commercially available turtle pellets, earth worms, and small fishes (*Osmerus eperlanus*). Animal keeping and experiments were in strict accordance with the Austrian Protection of Animals Act and the study was approved by the University of Vienna Advisory Board of Study Affairs. For morphological investigations, all six animals were anesthetized by intraperitoneal injection of sodium pentobarbital and, after deep narcosis, decapitated. The heads were immersed immediately in the fixation solutions described below.

For scanning electron microscopy (SEM), the heads of two juveniles were immersed for 24 h at room temperature in modified Karnovsky’s solution (2.5% glutaraldehyde and 2% formaldehyde in 0.1 M cacodylate buffer [30]). After rinsing in 0.1 M cacodylate buffer, the lower jaw with the tongue was removed from the head. Then, samples were postfixed in buffered 1% osmium tetroxide for 2 h at 37°C, washed in distilled water, and treated with 25% HCl at 40°C for 30 min to remove the mucus from the surface. After repeated rinsing in distilled water, the samples were dehydrated in a graded ethanol and acetone series and immersed in HMDS (hexamethyldisilazane) for seven days. Then, the HMDS was evaporated and the samples were mounted on aluminium stubs for SEM and coated with gold in an AGAR B7340 Sputtercoater (Agar Scientific Ltd, Stansted, UK). Observations and digital photographic documentations were made using a Philips XL-20 scanning electron microscope (Philips, Eindhoven, NL) and a Philips XL-30 environmental scanning electron microscope (Philips, Eindhoven, NL).

To analyze the “in situ” 3-D structure of the hyolingual skeleton, micro-CT (micro computed tomography) scans were performed on two individuals. They were anesthetized as described above, decapitated, and the heads were immersed in buffered 4% formaldehyde solution for three weeks, changing the solution once a week. Before scanning, the two heads were transferred in 70% alcoholic solution, which was changed three times. The CT-scans were performed by using a Sub-µm-device Nanotom (Phönix|x-ray, Wunstorf, GER). During measurement, projection images were grabbed using an amorphous Silicon matrix detector at several angular positions. After a full 360° rotation, 1500 images were generated. The images were reconstructed using the software provided with the micro-CT system. Gray values corresponding to the tissue density were assigned to each spatial element (voxel). The length of each voxel was 6 µm.

For 3-D-reconstruction and visualization, the resulting gray-scale image stacks were imported into Amira 4.1 software (Mercury Computer Systems, Chelmsford, MA, USA). Surfaces were generated as previously described by Heiss et al. [18].

For paraffin-based histology, two heads were immersed in Bouin’s fixative [31] for 50 days, changing the solution once a week. After complete fixation and decalcification, the upper jaw was removed from the rest of the head and the cornified rhamphothecae (“beak”) were cut off. Then, the samples were dehydrated in a graded ethanol-isopropanol series and embedded in paraffin. After polymerization, 7 µm serial-sections were made on a Reichert-Jung 2030 rotatory microtome (Reichert-Jung, Bensheim, GER). The sections were mounted on glass slides and, after removing the paraffin, stained with either Haematoxylin (H) and Eosin (E), periodic acid Schiff (PAS), Alcian blue (AB), or Coomassie Brilliant blue (after Romeis [31] and Kiernan [32]). As controls for the Coomassie Brilliant blue staining, we used sections from *Pleurodeles* skin (for details, see [33]). The preparations were documented by digital photography under a Nikon Eclipse E800 light microscope (Nikon, Tokyo, JP).

The nomenclature of the muscles described in the present study is mainly based on the terminology proposed recently by Ingmar Werneburg [34].

## Results

### Scanning Electron Microscopy

**Figure 1:**
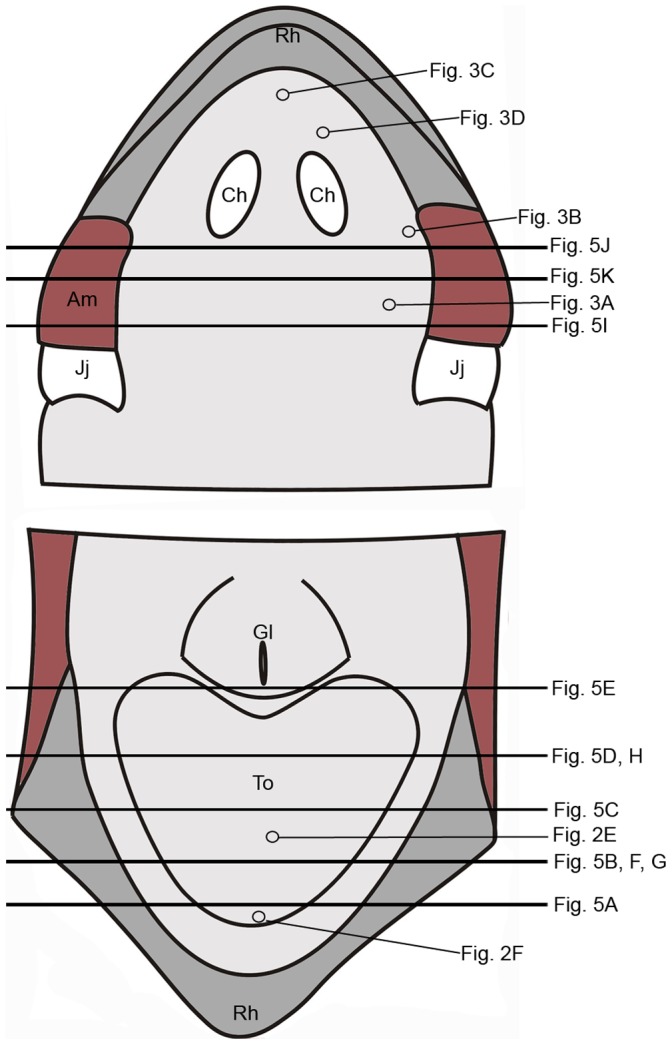
Schematic drawing showing palate (top) and floor of mouth (bottom) in *H grandis*. Circles indicate positions of SEM and horizontal lines positions of LM micrographs in figures 3–5. Am, adductor mandibulae (sectioned horizontally); Ch, choanae; Gl, glottis; Jj, jaw joint; Rh, rhamphotheca; To, tongue.

**Figure 2:**
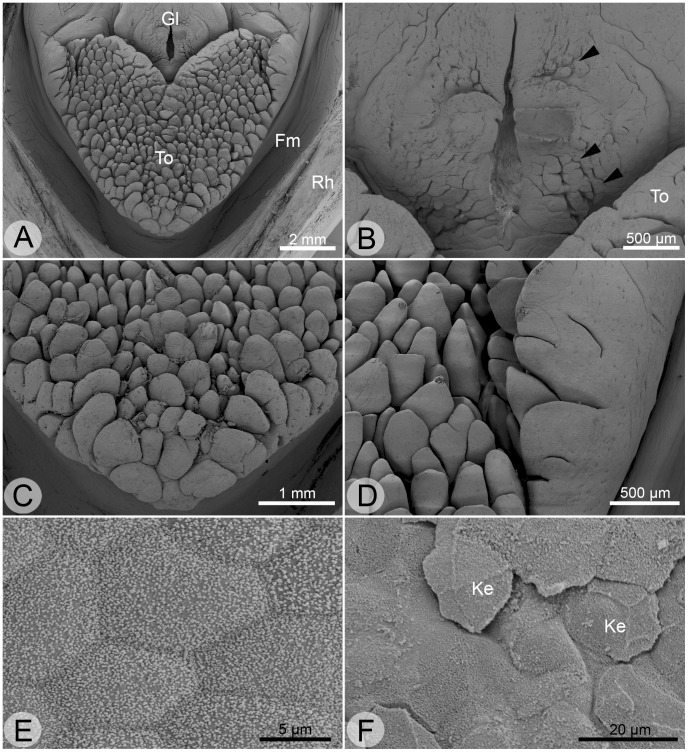
Scanning electron micrographs of the tongue. **A** overview of floor of mouth (Fm) with tongue (To), glottis (Gl) and a part of the lower rhamphotheca (Rh). **B** Higher magnification of glottis. Note the small papillae around the glottal slot (indicated by arrowheads). **C** Higher magnification of tongue tip and **D** of lateral tongue. Note the differences in papillae morphology. **E** apical epithelial surface of papillae is usually unkeratinized and studded with microvilli (visible as small white dots) but bears keratinocytes (Ke) on the tongue tip (**F**).

The floor of the mouth in *H. grandis* was anteriorly (“directed to the front”) and laterally (“directed to the sides”) marginated by the ventral horny beak and, posteriorly (“directed rearwards”), at the level of the glottis, it passed into the pharyngeal area (Fig. 1). The floor of the mouth was largely occupied by the heart-shaped tongue (see Fig. 1, Fig. 2A). The apex of the tongue and the two posterior horns were strongly rounded. The two posterior horns enclosed the glottal bulge and passed into the pharyngeal area (Fig. 2A, B). The surface of the tongue itself was studded with lingual papillae, which were bulky and blunt on the margins and became thinner towards the center (Fig. 2C, D). The surface of the lingual papillae was generally smooth, and higher magnification revealed that its cells were studded with microvilli (Fig. 2 E). Only the papillae on the very anterior tongue tip showed a slightly rough surface due to keratinocytes there (Fig. 2 F). Much smaller papillae than the lingual ones were present on the glottal bulge, around the glottal slot (Fig. 2 B). The surface of the pharyngeal area was rather smooth and unkeratinized, only occasionally showing longitudinally oriented grooves.

**Figure 3:**
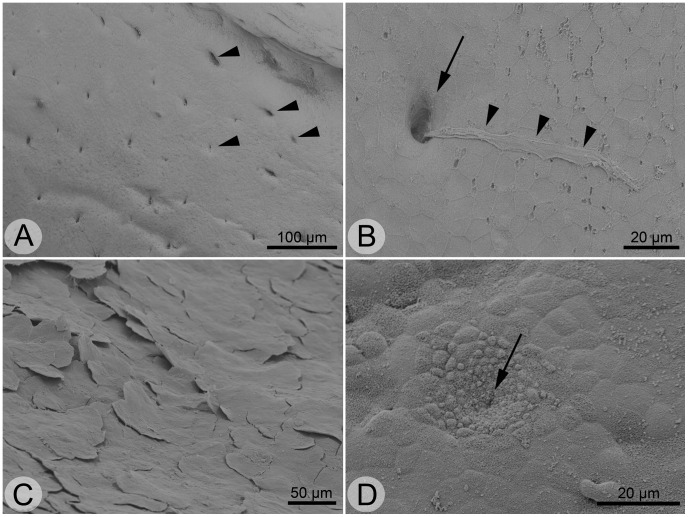
Scanning electron micrographs of the palate. **A** detail of posterior palate with numerous small gland pores opening into the oropharyngeal cavity (indicated by arrowheads). **B** detail of larger gland pore (arrow) with protruding mucus remnants (arrowheads). **C** keratinized epithelium from anterior (prechoanal) palate with flat keratinocytes. **D** detail of taste bud. Note the concentrically arranged small epithelial cells around the taste pore (arrow).

The palate was laterally and anteriorly marginated by the dorsal horny beak. The surface of the palate was rather smooth and between the lateral margins it bore the two slightly ellipsoid openings of the choanae (schematically shown in Fig. 1). Posteriorly, the palate passed into the pharyngeal area. The intersection was marked by the small openings of the eustachian tubes. Higher magnification revealed that the palatal mucosa was unkeratinized between and posterior to the choanae, where numerous glandular pores were present (Fig. 3 A, B), but keratinized in the anteriormost (“prechoanal”) palate (Fig. 3 C).

Taste buds were detected on the oropharyngeal surface based on their concentrically arranged epithelial cells and the taste pore in the center (Fig. 3 D). Taste buds were spread throughout the oropharyngeal cavity, but were mostly concentrated in the anterior and lateral floor of the mouth, the anterior palate (between horny beak and choanae), and on the lateral areas.

### Computed Tomography

**Figure 4:**
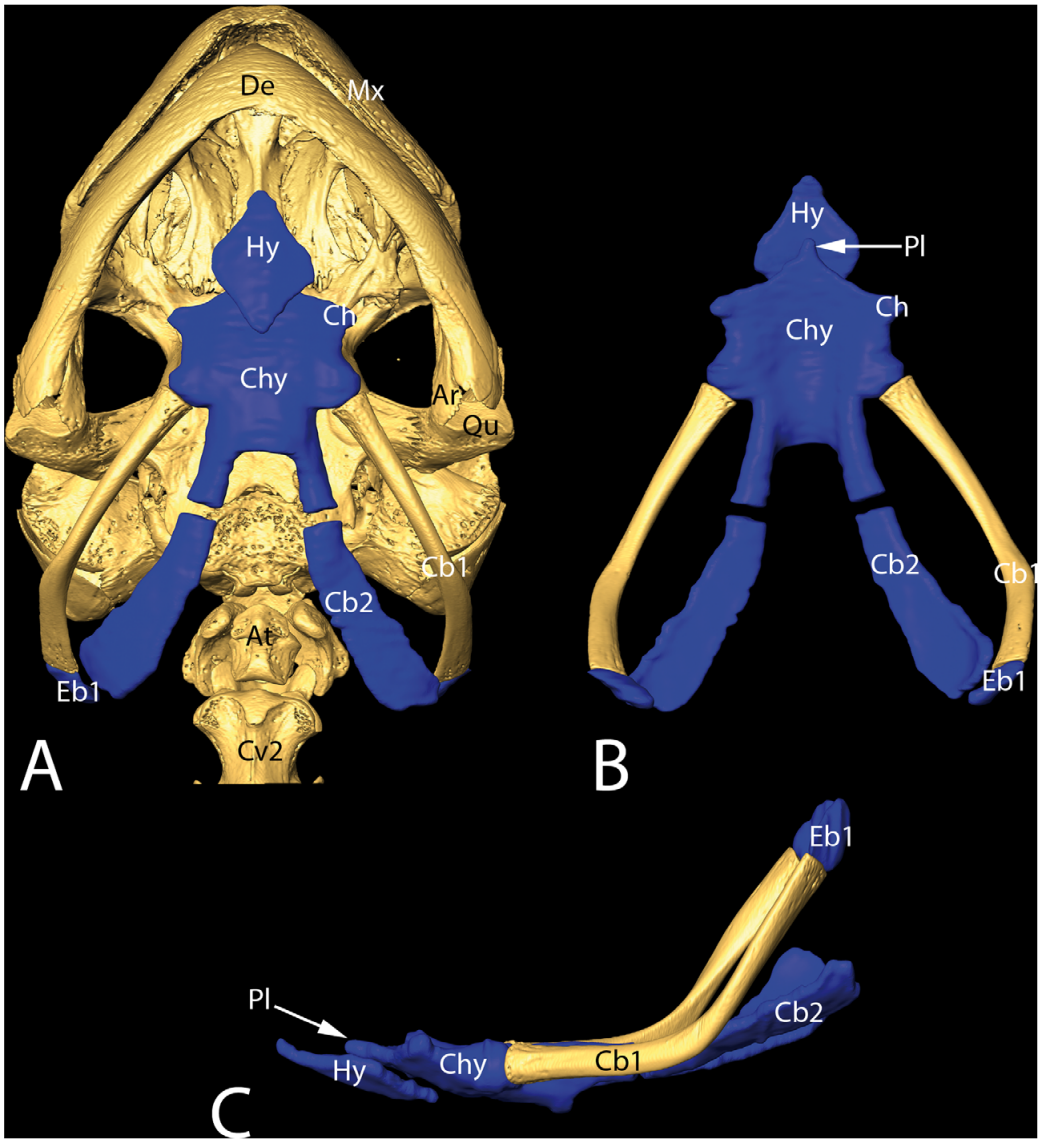
3-D reconstructions of skull and hyolingual skeleton in *H. grandis* based on micro-CT scans. A ventral view showing “in situ” position of the hyolingual skeleton. **B** dorsal and **C** lateral view after virtually removing the skull. Ar, articulare; At, atlas (first cervical vertebra); Cb1, ceratobranchial 1; Cb2, ceratobranchial 2; Ch, cornu hyale; Chy, corpus hyoidei; Cv2, second cervical vertebra; De, dentary; Eb1, epibranchial 1; Hy, hypoglossum; Mx, maxilla; Pl, processus lingualis; Qu, quadratum.

The hyolingual skeleton in *H. grandis* lay in the floor of the oropharynx (Fig. 4 A) and showed two posterior extensions, the branchial horns that ran further dorsolaterally and posteriorly into the pharyngo-esophageal region (Fig. 4 A–C). The hyolingual skeleton consisted of the cartilaginous main body (“corpus hyoidei” or “basihyal”), which showed one broad and blunt anterior protrusion, the lingual process (“processus lingualis”) (Fig. 4 B, C). Directly behind the lingual process lay, ambilaterally, the small hyoid horn (“cornu hyale”) (Fig. 4 A, B), which was followed posteriorly by the articulations with the first and second branchial horns (“cornua branchiales 1 and 2″) (Fig. 4 A–C). The first branchial horn consisted of a proximal large bony element, the ceratobranchial 1, and a distal small cartilaginous element, the epibranchial 1 (Fig. 4 A–C). The second branchial horn consisted only of the cartilaginous, laterally broadened ceratobranchial 2 (Fig. 4 A–C). The rhombus-shaped cartilaginous hypoglossum with slightly longitudinal elongation was located antero-ventrally to the lingual process of the corpus hyoidei (Fig. 4 A–C). The hypoglossum represented the anteriormost element of the hyolingual skeleton.

### Light Microscopy

#### The floor of the mouth

The floor of the mouth in *H. grandis* was composed by the oral mucosa, the deeper lying hyolingual skeleton (described above), and several muscles (described below).

The oral mucosa consisted of the mainly unkeratinized mucosal epithelium, which was supported by the lamina propria. The mucosal epithelium itself was rather thick and consisted of 6–8 cell layers. We detected mucosal keratinization only in regions very close to the rhamphotheca. The mucosal epithelium was separated proximally from the lamina propria by the basement membrane, a thin acellular layer. The mucosa of the floor of the mouth itself was further supported by several throat muscles and the hyolingual skeleton.

**Figure 5:**
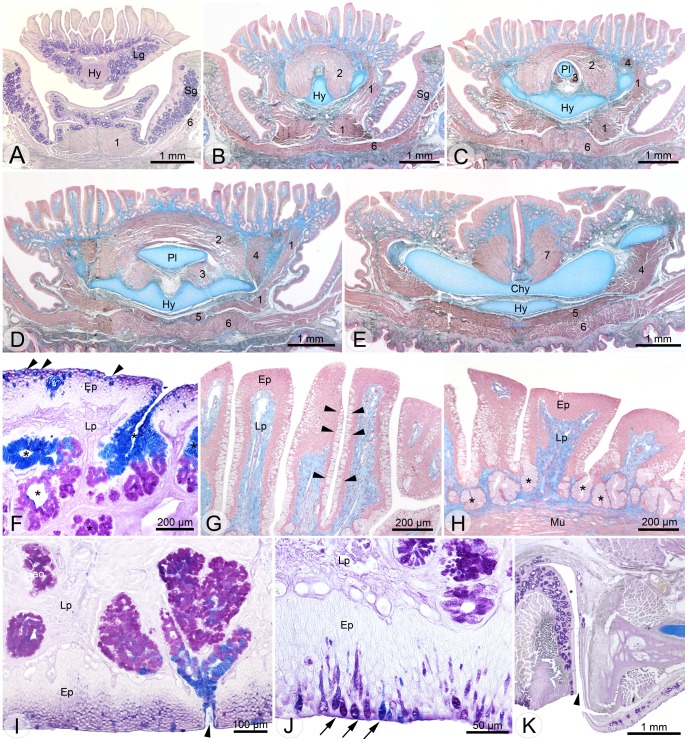
Light micrographs showing histological cross sections through the oropharynx in *H. grandis*. A –**E** overviews showing floor of mouth and tongue from anterior (**A**) to posterior (**E**). Note the changes in the sublingual and lingual glands, hyolingual skeleton and muscles. Muscles: (1) M. genioglossus, (2) M. hypoglossoglossus, (3) M. hypoglossohyoideus, (4) M. hyoglossus, (5) M. geniohyoideus, (6) M. intermandibularis, (7) M. constrictor laryngis. **F** detail of floor of mouth showing single-celled glands (goblet cells, indicated by arrowheads) and branched tubular glands (asterisks). **G** and **H** details of lingual papillae from the middle tongue part (**G**) and from the posterior tongue (**H**). Note the lateral surface of the papillae studded with goblet cells (arrowheads) and the tubular glands between the papillae (indicated by asterisks in **H**). Mu, muscle. **I** and **J** details of the palate showing a branched tubular gland deeply invaginated into the lamina propria (Lp) in **I** and goblet cells embedded into the epithelium (Ep) (indicated by arrows in **J**). **K** overview of the *glandula anguli oris*. Branched tubular glands open into a common duct (asterisk) that opens into the oral cavity (arrowhead). Chy, corpus hyoidei; Ep, epithelium, Hy, hypoglossum; Lg, lingual gland; Lp, lamina propria; Pl, processus lingualis; Sg, sublingual gland. Stainings: A: PAS; B–E, G, H: Azan; F, I–K: AB-PAS.

The ventalmost muscle, directly underlying the skin of the throat, was the M. intermandibularis, which ran transversely between the halves of the lower jaw (Fig. 5 A–E). It originated medially from the dentary, and both controlateral parts were connected by a medial aponeurosis (raphe medialis), which represented the insertion site. The deeper-lying M. geniohyoideus Pars lateralis (Fig. 5 D, E; 6 A, B) ran from the proximal part of the first branchial horn anteriorly to the hypoglossum and M. genioglossus (M. genioglossus will be described below as “tongue muscle”).

**Figure 6:**
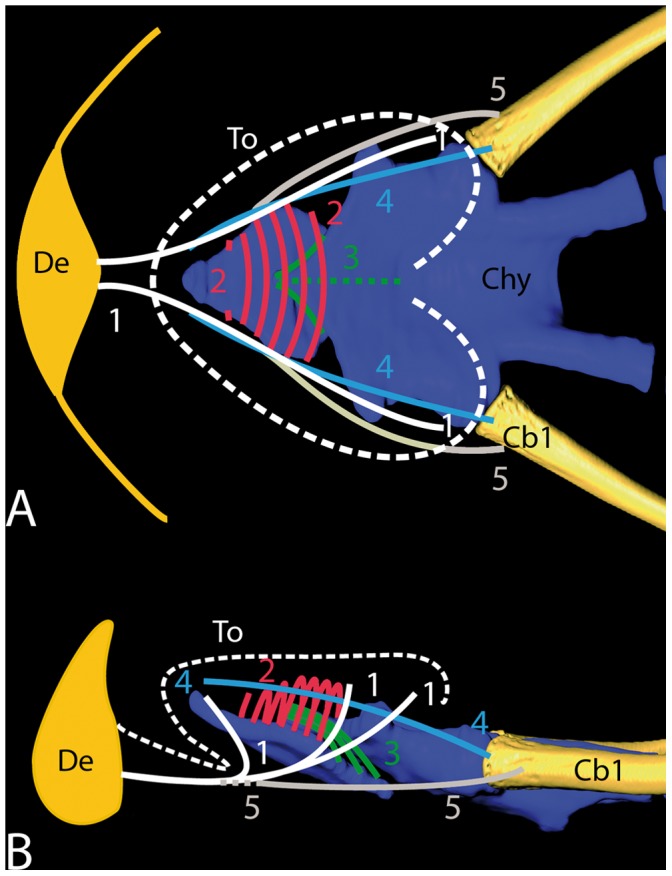
Anterior part of the hyolingual skeleton (based on 3-D reconstructions shown in Fig. 4) in A dorsal and B lateral view, with courses of the main lingual muscles (colored lines). (1, white) M. genioglossus; (2, red) M. hypoglossoglossus; (3, green) M. hypoglossohyoideus; (4, blue) M. hyoglossus; (5, grey) M. geniohyoideus. Cb1, Ceratobranchial 1; Chy, corpus hyoidei; De, dentary; To, tongue (white dashed line).

Mucosal glands were numerous in the floor of the mouth and were present as discrete goblet cells embedded in the epithelium, goblet cell aggregations, or glands organized to single tubular or branched tubular entities (Fig. 5 A–F). The largest glandular entity was the sublingual gland. It consisted of densely packed, branched tubular glands. The sublingual gland was positioned posterior to the mandibular symphysis and split early into a left and a right part, which ran further posteriorly medial to the mandibles and enclosed the tongue laterally. All glands from the floor of the mouth showed positive reaction to Alcian blue (AB) at pH 2.5 (for acid mucosubstances), PAS (for neutral mucosubstances), or both AB and PAS (Fig. 5 F), but negative reaction to Coomassie Brilliant blue (for proteins; controls reacted positively). If stained with both AB and PAS, simple tubular and branched tubular glands showed a staining dichotomy, with both AB- and PAS-positive cells proximally and AB-positive cells distally (Fig. 5 F, I).

For details on the hyolingual skeleton, see previous section on CT.

#### Tongue

The tongue was covered by the lingual mucosa that showed keratinization only in the dorsal epithelium of the anteriormost lingual papillae. The lingual papillae were restricted to the dorsal tongue face. While their apices were covered by cuboidal and stratified epithelium, their shafts showed numerous goblet cells that increased in number proximally to the openings of the tubular gland invaginations between the papillae (Fig. 5 G, H). Taste buds were present on the tongue surface, especially embedded in the lateral epithelium of the papillae. They were distributed randomly, with no clear pattern.

The musculature associated with the tongue was well developed in *H. grandis*. The M. genioglossus originated from the dentary bone, lateral to the mandibular symphysis, and ran posterolaterally for the entire length of the tongue. Anteriorly, the fibers of the M. genioglossus fanned out vertically and inserted on the dorsal mucosa of the tongue tip. More posteriorly, the fibers became oriented longitudinally, forming the muscular sides of the tongue (Fig. 5 A–D; 6 A, B). The M. hypoglossoglossus originated laterally on the anterior half of the hypoglossal cartilage and ran upwards. Anteriorly, the muscle fibers of the right and left portion of the M. hypoglossoglossus ran upwards to insert on the dorsal mucosa of the tongue. More posteriorly, the fibers of the right and left portion were curved to the midline, where they met and formed an arch-like myostructure, enclosing the lingual process of the hyoid body and the M. hypoglossohyoideus there (Fig. 5 B–D; 6 A, B). The M. hypoglossohyoideus originated laterally on the distal portion of the lingual process and ran caudoventrally to insert dorsally on the hypoglossal cartilage (Fig. 5 C, D; 6 A, B). The M. hyoglossus originated on the proximal part of the first branchial horn, and ran anterodorsally, dorsal to the hypoglossum to insert on the dorsal lingual mucosa of the anterior tongue (Fig. 5 C–E). The M. hyoglossus lay between M. hypoglossoglossus, M. genioglossus, and dorsal lingual mucosa. No branchings of any lingual muscle fibers entering the lingual papillae were observed in our material.

#### Palate

The palatal mucosa was comparable to the general pattern described above but varied in thickness. In the area anterior to the choanae, it separated the oral from the nasal cavities and was thick and strongly keratinized. No keratinization was observed further posterior to this region (except very close to the rhamphotheca). A few mucosal glands in the “prechoanal” palate were represented only by discrete goblet cells; no glandular invaginations were observed there. The palatal mucosa near the choanae and at further posterior part was slightly wider. In this region, we also documented the first glandular invaginations (i.e. tubular glands) besides goblet cells (Fig. 5 I, J). Posterior to the choanae, oral glands became more numerous, increased in size and complexity, and formed a dense glandular field that extended widely posteriorly. In the corner of the mouth a distinct and large gland, the *glandula anguli oris*, was detected (Fig. 5 K). The *glandula anguli oris* was deeply embedded between adductor muscle, eye, and the connective tissue of the roof of mouth; it opened through a single duct into the oral cavity. All palatal glands showed the same histochemical reaction as the sublingual and lingual glands (see respective sections above and Fig. 5 F, I–K).

## Discussion

Extant turtles show adaptations to a great diversity of ecological demands, spanning the range from fully aquatic to fully terrestrial forms. This diversity is largely congruent with phylogeny and reflected in structural specializations of the oropharynx [16], [17], [20], [22]–[26], [35]. The oropharynx plays a key role in tetrapod adaptation processes and is involved in a variety of crucial functions, ranging over aquatic/terrestrial respiration, thermoregulation, olfaction, defence, display, and, of course, feeding [4].

Turtles split from other amniotes over 220 Ma (million years) ago. Even if the oldest known fossil turtle record, *Odontochelys semitestacea*, was probably a marine animal [36], the question whether turtles evolved from an aquatic or a terrestrial ancestor is still under discussion [37]. This discussion is further complicated by the fact that turtles early in their history branched into aquatic, semiaquatic, and terrestrial forms. Nonetheless, it is generally accepted that the ancestor leading to the lineage of extant turtles was aquatic [3], [38]. Consequently, the terrestrial evolution in extant turtles proceeded parallel and independently to other amniotes [18]. The three extant turtle families displaying terrestrial lifestyles have evolved into partly (emydids and geoemydids) or fully (testudinids) terrestrial forms independently from each other.

The geoemydid *H. grandis* studied here shows a semiaquatic to terrestrial lifestyle, and we hypothesized that the parallel adaptation of its terrestrial trophic biology is reflected by its oropharyngeal morphology. This was predicted based on the assumption that the functional demands on the feeding biology (and therefore on the oropharyngeal system) between water and air are different and to a large degree conflicting [39]. In fact, *H. grandis* shows adaptations to terrestrial food uptake, even if still able to feed in water. These adaptations show certain parallels to those in purely terrestrial turtles, the testudinids [10], [14], [16], [18]. Analogous to testudinids, the Giant Asian pond turtle has a large muscular tongue studded with large lingual papillae. Such papillae increase the lingual surface and therefore the friction force between tongue surface and food – advantageous if the tongue is used for intraoral food transport on land. Unlike in testudinid turtles, however, the lingual mucosa in *H. grandis* is poorly keratinized, occasionally showing slight keratinization only on the very anterior tongue tip. An unkeratinized tongue surface is typically found in turtles strongly associated with aquatic environments [23], [24], [26]. Similar to testudinids, the tongue in *H. grandis* is very movable and can be protracted out of the mouth and retracted again to transport a grasped food item back to the esophagus for further swallowing [40]. To perform such movements, *H. grandis* needs a specialized hyolingual skeleton with well-developed lingual muscles. The principal components of the hyolingual skeleton are more or less the same in all extant turtles studied so far [12], [18], [34], [41]–[46]. Nonetheless, the extension and composition of elements are considerably different between species. The main component is the central hyoid body, which articulates with the paired first and second branchial horns. Anteriorly, directly beneath the hyoid body, lies the hypoglossum, a hyolingual element unique for – and found in all – turtles. To manage the biophysical demands of food uptake underwater (i.e. compensatory or inertial suction feeding; see [5]), aquatic turtles have prominent posterior hyolingual elements, and the hyolingual skeleton as a whole is typically ossified [43]. These structural enforcements are needed to handle the strong forces developed by the massive muscles that retract the hyolingual complex; this retraction explosively expands the oropharyngeal cavity and creates suction. Terrestrial turtles, in contrast, do not rely on suction feeding but need a flexible support for the tongue; here, the anterior hyoid components are generally enlarged and the hyolingual skeleton remains mainly cartilaginous. The hyolingual skeleton in *H. grandis* shows such adaptations to functional terrestrial feeding demands and is thus similar to testudinids. In contrast, however, the anterior hyolingual components (i.e. the lingual process and the anterior hypoglossal process) are smaller [10], [18], [41]. Besides supporting the floor of the mouth and tongue, the hyolingual skeleton provides insertion sites for lingual muscles and other muscles associated with the mechanics of food uptake. The main “intrinsic and extrinsic” (for an in-depth discussion on the dichotomy of lingual muscles see [47]) lingual muscles are apparently present in most turtles (for a possible exception see [43]), and their contribution to lingual movements was recently described for the primitive testudinid *Manouria emys* [18].

The mobility of the tongue as a unit is widely coupled to the movement of the hyolingual skeleton as a whole [1]. The first step in tongue protraction was suggested by Heiss et al. [18] to involve protraction of the entire hyolingual skeleton by contraction of the M. geniohyoideus which inserts to the M. genioglossus that runs to the dentaries. In a second step, contraction of the M. hypoglossohyoideus, which runs between the anterior part of the lingual process and posterior hypoglossum, compresses these skeletal structures relative to each other. This causes rostral sliding of the hypoglossum relative to the processus lingualis of the hyoid, resulting in further tongue elongation. This movement is probably stabilized and further supported by contraction of the medial and posterior M. hypoglossoglossus. The M. hypoglossoglossus originates on the lateral parts of the hypoglossum and, anteriorly, its fibers are curved to the midline; this forms a muscular arch which encircles both lingual process and the M. hypoglossohyoideus. Contraction of the arch-shaped hypoglossoglossus muscle fulfils two functions: it stabilizes hypoglossal sliding (caused by the M. hypoglossohyoideus) and may also support this movement by pressurizing the lingual process, causing the hypoglossum to slide anteriorly along the lingual process.

For tongue retraction, it was suggested that contraction of the hyoglossus muscle, which runs between tongue tip and proximal first branchial horn, causes retraction of the hypoglossum relative to the lingual process. Further tongue retraction might be caused by retraction of the whole hyolingual skeleton by the coracohyoideus muscle, which runs between the hyolingual skeleton and shoulder girdle. The principal lingual myoskeletal architecture in *H. grandis* is comparable to that in testudinids. Accordingly, a similar lingual function, especially regarding intraoral food transport, is expected, as has been previously hypothesized for semiaquatic turtles in general [18]. Contrary to testudinids, however, *H. grandis* is still able to feed underwater. This is probably because it can create a certain amount of suction – in principle similar to aquatic turtles – by rotating the hyolingual system posteroventrally [6], [9], [42], [48], [49], [50]. Consequently, its posterior hyolingual elements are clearly enlarged compared to testudinids, but still relatively small compared to aquatic specialists [42], [43].

Analogous to the hyolingual myo-skeletal architecture, the design of the oropharyngeal glands correlates highly with phylogeny, habitat and possibly diet in turtles [14], [15], [17], [20], [26], [50]. Aquatic turtles show simple single-celled glands (goblet cells) in their oropharyngeal cavity [16], [19]–[25], whereas terrestrial turtles have complexly arranged and prominent oropharyngeal glands [10], [14]–[16], [18], [20]. In this respect, *H. grandis* shows some parallels to purely terrestrial turtles. Its sublingual and palatal glands, as well as the *glandula anguli oris* represent the complex morphotype (branched and compound tubular glands) so far only known for testudinids. On the other hand, goblet cells are still scattered throughout the oropharynx in *H. grandis* – typical features for aquatic turtles. This indicates a lower degree of terrestrial specialization in *H. grandis* compared to testudinids. The prevalence of goblet cells and only few tubular glands were previously described for another semiaquatic geoemydid that, in turn, shows a much higher affinity to water: *Cuora amboinensis* [50].

Similar to other turtles, the oropharyngeal glands in *H. grandis* produce and store mucus, as shown by their staining properties. The glands reacted positively to PAS and AB (for neutral and acidic mucopolysaccharides) but negatively to the Coomassie Brilliant blue test (for proteins). Nonetheless, proteinaceous material in the form of proteins associated with carbohydrates could also be present in small amounts, but was not detected in this study. Mucus plays an important role in terrestrial feeding biology. The lubricating nature of mucus helps avoid dehydration of the oropharyngeal epithelium [14]. It also improves the lingual-based terrestrial food ingestion and oropharyngeal transport because it makes the tongue “sticky” (see [1], [6]). Finally, mucus as a lubricating and “gliding” medium is fundamental for further swallowing in the terrestrial environment [1].

Similar to the increase of mucous glandular tissue, an increase in the keratinization of the oropharyngeal epithelium on the tongue, palate, and floor of the mouth is correlated with terrestrial feeding biology [17], [26]. A keratinized epithelium not only provides mechanical protection from abrasive food, but also serves as a “barrier” to avoid dehydration. Consequently, aquatic (non-marine-) turtles show keratinization only close to the rhamphothecae on the palate and floor of the mouth, sometimes accompanied by slight keratinisation on the tongue [21]–[24], [26]. *Heosemys grandis* represents an intermediate form in this respect, with a certain amount of keratinization on the tongue tip and anterior palate.

Taste buds as primary taste organs are also important in feeding because they enable the animals to discriminate between food items that have already been grasped and are being held between the jaws. The higher concentration of these organs in the anterior oral cavity (anterior palate, anterior floor of mouth) of *H. grandis* is congruent with its food-grasping mode, which is jaw based both in water and on land [40]. This allows a fast decision on accepting potential food and rejecting harmful items. Lingual based prehension modes are so far known only for more derived terrestrial turtles (i.e. “higher” testudinids).

In conclusion, the geoemydid turtle *H. grandis* shows a variety of morphological adaptations in its oropharynx for a terrestrial trophic biology – even if some primitive characters (typical for aquatic turtles) are still present. These adaptations to terrestrial feeding comprise the large tongue studded with large lingual papillae, the well-developed lingual muscles, complex oropharyngeal glands, and some oropharyngeal keratinization. We suggest that these adaptations occurred parallel to testudinids, the only exclusively terrestrial family among living turtles. Furthermore, the almost identical arrangement of the lingual myo-skeletal system in *H. grandis*, other geoemydids [12], and testudinids [10], [18] points to a general myo-skeletal pattern that was already present in the common ancestor of both families. Further detailed studies on emydids and out-groups from other turtle families should clarify whether this myo-skeletal architecture is conservative within the superfamily Testudinoidea, or within turtles in general.
